# Retraction Note: The effect of *Ganoderma lucidum* polysaccharide extract on sensitizing prostate cancer cells to flutamide and docetaxel: an in vitro study

**DOI:** 10.1038/s41598-025-34603-1

**Published:** 2026-01-06

**Authors:** Ramin Rahimnia, Mohmammad Reza Akbari, Alimohammad Fakhr Yasseri, Diana Taheri, Akram Mirzaei, Helia Azodian Ghajar, Parmida Dehghanpoor Farashah, Leila Zareian Baghdadabad, Seyed Mohammad Kazem Aghamir

**Affiliations:** 1 Department of Medical Nanotechnology, School of Advanced Technologies in Medicine, Tehran University of Medical Sciences, Tehran, Iran; 2 Women’s College Research Institute, Women’s College Hospital, University of Toronto, Toronto, Canada; 3 Institute of Medical Science, Faculty of Medicine, University of Toronto, Toronto, Canada; 4 Alborz University of Medical Sciences, Shariati Hospital, Alborz, Iran; 5 Isfahan Kidney Disease Research Center, Department of Pathology, Isfahan University of Medical Sciences, Isfahan, Iran; 6 Urology Research Center, Tehran University of Medical Sciences, Tehran, Iran

Retraction of: *Scientific Reports* 10.1038/s41598-023-46118-8, published online 02 November 2023

The Editors have retracted this Article.

After publication, concerns were raised regarding the data presented in the Figures, specifically:

- Fig. 1 PC3 10um Doc appears to overlap with Fig. 1e of^1^;
- Fig. 1 LNCap 5um Doc and Flu-Doc images appear to overlap;
- Fig. 3A and B share similar features, and C and D appear highly similar;
- Fig. 4A LNCap 5µM Doc and LNCap Flu, Doc & Ganoderma lucidum plots appear highly similar;
- Fig. 4A PC3 10µM Doc and PC3 Doc + Ganoderma lucidum plots appear highly similar.
- A number of flow cytometry plots in Fig. 4A appear higly similar to those in Figs. 3 and 4 of^1^ and Fig. 5 of^2^, representing different cell lines and treatments;
- Fig. 6A 3 combination and 6B Flu+Doc images appear highly similar;
- Fig. 6A 5µM Doc and 6B 10µM Doc images appear to overlap;
- Fig. 6A 20µM GLP and 6B Doc+GLP images appear to overlap;
- Fig. 6A Flu+GLP and 6B Control-0 images appear to overlap with rotation;
- Fig. 7 20µM Flu, Flu+GLP and 3 combination images appear to originate from the same sample;
- Fig. 7 10µM Doc and 30µM GLP appear highly similar (with different brightness and contrast).

The Editors therefore no longer have confidence in the presented data.

Seyed Mohammad Kazem Aghamir agrees with this retraction. The other Authors have not responded to any correspondence from the Editors about this retraction.
